# Depressive symptoms and multiple markers of brain aging in community-dwelling older adults

**DOI:** 10.3389/fnagi.2026.1868746

**Published:** 2026-06-23

**Authors:** Jea Chul Ha, Jiyoun Jung, Yu-Mi Kim, Mi Kyung Kim, Min-Ho Shin, Sang Baek Koh, Hyeon Chang Kim, In-Sung Chung

**Affiliations:** 1 Department of Occupational and Environmental Medicine, Keimyung University School of Medicine, Daegu, Republic of Korea; 2 Department of Occupational and Environmental Medicine, Keimyung University Dongsan Hospital, Daegu, Republic of Korea; 3 Department of Preventive Medicine, Hanyang University College of Medicine, Seoul, Republic of Korea; 4 Department of Preventive Medicine, Chonnam National University Medical School, Hwasun, Republic of Korea; 5 Department of Preventive Medicine, Institute of Occupational Medicine, Yonsei Wonju College of Medicine, Wonju, Republic of Korea; 6 Department of Preventive Medicine, Yonsei University College of Medicine, Seoul, Republic of Korea

**Keywords:** brain aging, cognitive performance, depressive symptoms, hippocampal volume, white matter hyperintensities

## Abstract

Late-life depressive symptoms have been associated with structural brain changes and cognitive impairment, but prior studies have typically examined individual markers of brain aging in isolation. This study examined the associations between depressive symptoms and multiple markers of brain aging in a population-based cohort of community-dwelling older adults. Baseline data from 2,746 participants with complete data on depressive symptoms, neuroimaging measures, cognitive assessment, and covariates were analyzed cross-sectionally. Depressive symptoms were assessed using the Center for Epidemiologic Studies Depression Scale (CES-D). White matter hyperintensity (WMH) volume and hippocampal volume were derived from structural magnetic resonance imaging, and global cognitive performance was assessed using the Seoul Neuropsychological Screening Battery-Core (SNSB-C). Multivariable linear regression models were used to evaluate associations after adjustment for demographic, cardio-metabolic, and lifestyle factors. Higher depressive symptom burden was associated with greater WMH burden, smaller hippocampal volume, and poorer global cognitive performance. Associations with WMH burden and cognitive performance remained consistent after additional adjustment for vascular comorbidities and in sensitivity analyses modeling depressive symptoms as a dichotomous exposure using a CES-D cutoff of ≥16, whereas associations with hippocampal volume were more modest. These findings suggest that depressive symptoms in later life are related to multiple domains of brain aging, with the most consistent associations observed for WMH burden and cognitive performance. Further longitudinal studies are needed to clarify temporal relationships underlying these associations.

## Introduction

Late-life depressive symptoms are common among community-dwelling older adults and represent a major public health concern because of their association with functional decline, reduced quality of life, and increased risk of adverse neurological outcomes (Alexopoulos, 2019; Livingston et al., 2020). Beyond their psychiatric impact, depressive symptoms in later life have been increasingly linked to structural brain changes and cognitive impairment, suggesting that they may be related to broader aspects of brain aging rather than solely reflecting mood disturbance (Alexopoulos, 2019; Diniz et al., 2013; Debette and Markus, 2010; Herrmann et al., 2008; Taylor et al., 2013; Alexopoulos et al., 1997; McEwen, 2007).

A substantial body of research has examined potential mechanisms underlying this relationship. One line of work has emphasized cerebrovascular pathology, particularly white matter hyperintensities (WMHs), as markers of cerebral small vessel disease associated with late-life depressive symptoms (Debette and Markus, 2010; Herrmann et al., 2008; Taylor et al., 2013). This has led to the vascular depression hypothesis, in which vascular injury disrupts fronto–subcortical circuits involved in mood regulation (Taylor et al., 2013; Alexopoulos et al., 1997). In parallel, other studies have focused on neurodegenerative processes, with evidence suggesting associations between depressive symptoms and reduced hippocampal volume as well as stress-related neurobiological changes (McEwen, 2007; Campbell et al., 2004; Videbech and Ravnkilde, 2004). In addition, depressive symptoms have been associated with poorer cognitive performance and an increased risk of cognitive decline, further supporting their potential relevance to brain aging in older adults (Livingston et al., 2020; Diniz et al., 2013).

Despite these findings, prior studies have typically examined individual markers of brain aging in isolation, focusing separately on vascular injury, structural neuro-degeneration, or cognitive function. However, brain aging is inherently multidimensional, and depressive symptoms in later life may reflect the influence of multiple underlying processes rather than a single pathway. Evaluating these domains together within the same population-based cohort may therefore provide a more comprehensive understanding of how depressive symptoms relate to brain aging in later life. Furthermore, population-based neuroimaging studies addressing these relationships in non-Western cohorts remain limited, constraining the generalizability of existing evidence.

Therefore, the present study aimed to examine the association between depressive symptoms and multiple markers of brain aging in a community-based cohort of older adults. Specifically, we investigated whether depressive symptoms were associated with WMH burden as an indicator of cerebrovascular injury, hippocampal volume as a structural marker of neuro-degeneration, and global cognitive performance. By evaluating these domains together within a single population-based neuroimaging cohort, this study sought to provide an integrated perspective on the relationship between depressive symptoms and multidimensional aspects of brain aging in later life.

## Materials and methods

### Data source and study design

The present study used baseline data from the Cognitive Aging (CA) subcohort of the Cardiovascular Disease Association Study (CAVAS), a component of the Korean Genome and Epidemiology Study (KoGES) (Kim et al., 2017; Lee et al., 2025; Namgung et al., 2023). KoGES is a nationwide community-based research program supported by the Korea National Institute of Health that aims to identify genetic, environmental, and lifestyle determinants of chronic diseases in the Korean population. The CAVAS component of KoGES was initiated in 2005 to investigate the burden of cardiovascular diseases and related health disparities between urban and rural communities across South Korea.

The CAVAS-CA subcohort was established to examine factors associated with healthy aging and cognitive function. Participants aged 55 years or older were recruited from five regional study sites in South Korea. Baseline enrollment for the CAVAS-CA cohort was conducted between 2020 and 2022 using standardized assessment protocols implemented across all study centers.

The baseline examination included psychosocial questionnaires, clinical evaluations, detailed neuropsychological testing, and structural brain magnetic resonance imaging (MRI). Follow-up assessments are planned at approximately three-year intervals as part of the ongoing longitudinal investigation in the CAVAS-CA cohort; however, follow-up data were not available for the present analysis. Accordingly, the present study used baseline data in a cross-sectional framework to examine associations between depressive symptoms and neuroimaging and cognitive markers of brain aging.

### Study participants

A total of 2,908 community-dwelling adults aged 55 years or older completed the baseline CAVAS-CA assessment and underwent brain MRI between 2020 and 2022. Participants were eligible for inclusion in the present analysis if they had valid MRI-derived measurements of WMH volume and hippocampal volume, completed the 20-item Center for Epidemiologic Studies Depression Scale (CES-D), underwent cognitive assessment using the Seoul Neuropsychological Screening Battery-Core (SNSB-C), and had complete data on intracranial volume and study covariates.

**Figure 1:**
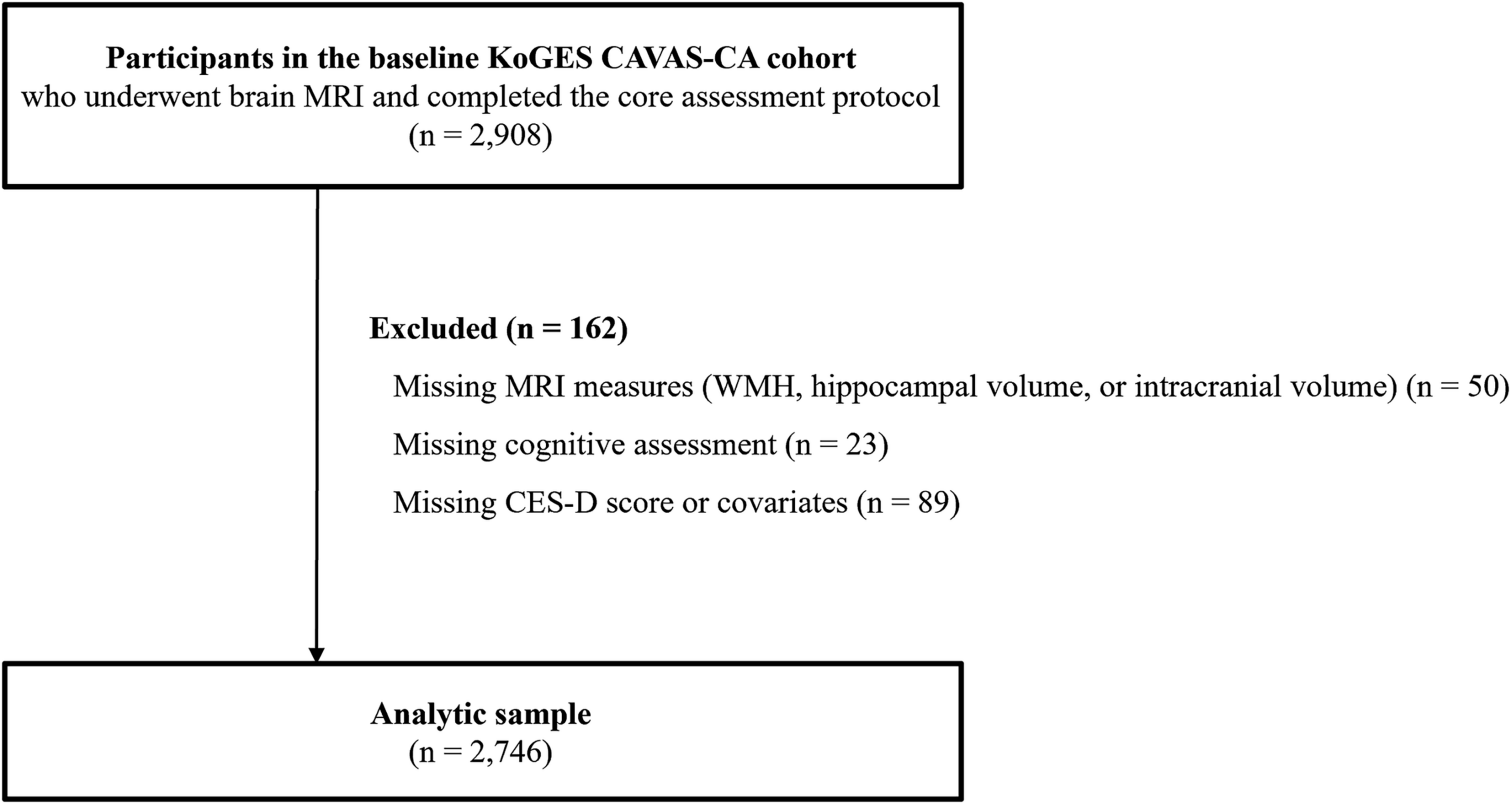
Participant selection flow diagram. KoGES CAVAS-CA, Korean Genome and Epidemiology Study Cardiovascular Disease Association Study–Cognitive Aging; CES-D, Center for Epidemiologic Studies Depression Scale; WMH, white matter hyperintensity. The analytic sample included participants with complete data on depressive symptoms, neuroimaging measures, cognitive assessment, and pre-specified covariates.

After excluding participants with missing MRI measures (WMH volume, hippocampal volume, or intracranial volume; *n* = 50), missing cognitive assessment data (*n* = 23), and missing CES-D score or covariates (*n* = 89), 2,746 participants were included in the analytic sample. The participant selection process is presented in Figure 1.

### Measurements

#### Assessment of depressive symptoms

Depressive symptoms were assessed using the Korean version of the CES-D (Radloff, 1977; Cho and Kim, 1998). The CES-D is a widely used self-report instrument consisting of 20 items that assess the frequency of depressive symptoms experienced during the past week (Radloff, 1977). Each item is rated on a 4-point Likert scale ranging from 0 to 3, with higher values indicating greater symptom frequency.

In the Korean version, three positively worded items (items 5, 10, and 15) are reverse-coded prior to score calculation (Cho and Kim, 1993). The total CES-D score is calculated by summing all item scores, yielding a possible range of 0 to 60, with higher scores indicating greater depressive symptom severity.

The Korean version of the CES-D has been validated in previous studies and shown to have good reliability and diagnostic validity for depressive symptoms in community populations (Cho and Kim, 1998; Cho and Kim, 1993). In the present analytic sample, internal consistency was high (Cronbach’s *α* = 0.91).

For descriptive and regression analyses, CES-D scores were examined as a continuous variable and as quartiles based on their distribution in the analytic sample. In sensitivity analyses, depressive symptoms were also analyzed as a dichotomous exposure using a CES-D cutoff of ≥16 to indicate clinically relevant depressive symptom burden.

#### MRI acquisition and image processing

Structural brain MRI was performed at all study sites using 3.0 Tesla scanners with harmonized imaging protocols to ensure consistency across sites. Imaging platforms included Philips systems (Ingenia CX or Achieva dStream) and a Siemens Skyra scanner. MRI sequences included fluid-attenuated inversion recovery (FLAIR) imaging for the assessment of WMHs, along with high-resolution structural sequences for volumetric analyses.

Automated image processing was conducted using a standardized pipeline developed by the Hanyang CAVAS-MRI Quality Control Center. This pipeline included centralized image quality control, pre-processing of structural MRI data, automated lesion segmentation, volumetric extraction, and participant-level data verification before statistical analysis. WMHs were identified on FLAIR images using a convolutional neural network based on a U-shaped encoder–decoder architecture (U-Net), which has been widely applied for automated segmentation of WMH lesions (Ronneberger et al., 2015; Wu et al., 2019). Segmentation outputs were checked within the quality-control pipeline to identify gross processing errors or outlying values. The algorithm generated participant-level estimates of total WMH volume, expressed in cubic millimeters (mm^3^).

Hippocampal volumes were obtained from automated segmentation of structural MRI data. The same centralized processing framework was used to derive volumetric measures from high-resolution structural images. Volumes of the left and right hippocampus were summed to derive total hippocampal volume for each participant. Intracranial volume (ICV) was also estimated from structural MRI data and included as an adjustment variable in regression models to account for inter-individual differences in head size.

#### Cognitive assessment

Global cognitive performance was assessed using the SNSB-C, a standardized neuropsychological test battery widely used in Korea for the evaluation of cognitive function in older adults (Jahng et al., 2015; Ryu and Yang, 2023). The SNSB-C assesses multiple cognitive domains, including attention, language, visuospatial ability, memory, and executive function.

In the present study, global cognitive performance was represented by the composite total T-score derived from the SNSB-C. T-scores are standardized according to age and years of education based on normative data, with a mean of 50 and a standard deviation of 10 (Jahng et al., 2015). Higher T-scores indicate better cognitive performance.

The SNSB-C was administered by trained examiners using standardized procedures as part of the baseline CAVAS-CA assessment protocol.

### Covariates

Covariates were selected *a priori* based on established associations with depressive symptoms, vascular risk, and markers of brain aging. Demographic characteristics included age (years), sex (male or female), and years of formal education. Marital status was categorized as married versus not married.

Cardio-metabolic risk factors included body mass index (BMI; kg/m^2^) and systolic blood pressure (SBP), defined as the lower value of two seated measurements obtained during the clinical examination.

Lifestyle factors included smoking status and alcohol consumption. Smoking status was categorized as never, former, or current smoker based on self-reported smoking history. Alcohol intake was classified according to self-reported drinking frequency as none, ≤once per week, or >once per week.

In sensitivity analyses, additional vascular-related conditions were considered, including self-reported physician-diagnosed hypertension, diabetes mellitus, and dyslipidemia. These variables were included to evaluate the robustness of associations between depressive symptoms and brain aging markers after accounting for potential vascular comorbidities.

### Statistical analysis

Participant characteristics were summarized according to quartiles of the CES-D total score. Continuous variables were compared using one-way analysis of variance, except for WMH volume, which was compared using the Kruskal–Wallis test because of its skewed distribution. Categorical variables were compared using Pearson’s Chi-square tests. WMH volume was natural log–transformed prior to regression analyses.

Multivariable linear regression models with robust standard errors were used to examine the associations of depressive symptoms with WMH burden, hippocampal volume, and cognitive performance. Because these outcomes represent different domains of brain aging, they were analyzed separately using outcome-specific regression models. Depressive symptoms were analyzed both as a continuous variable, per 1-point increase in CES-D score, and as quartiles of the CES-D total score, with the lowest quartile serving as the reference category.

Three nested regression models were constructed to assess the stability of associations across levels of covariate adjustment. Model 1 was adjusted for age and sex. Model 2 was additionally adjusted for years of education and marital status. Model 3 was further adjusted for BMI, SBP, smoking status, and alcohol consumption. Hippocampal volume models additionally included intracranial volume to account for inter-individual differences in head size. For quartile analyses, overall differences across CES-D categories were evaluated using Wald tests, and linear trends across ordered quartiles were assessed by modeling CES-D quartiles as an ordinal variable.

Several sensitivity and supplementary analyses were conducted. First, models for WMH burden, hippocampal volume, and cognitive performance were additionally adjusted for self-reported physician-diagnosed hypertension, diabetes mellitus, and dyslipidemia. Second, depressive symptoms were also analyzed as a dichotomous exposure using a CES-D cutoff of ≥16 to indicate clinically relevant depressive symptom burden. Third, because the SNSB-C total T-score is standardized according to age and years of education, cognitive performance models were repeated after excluding age and education from covariate adjustment to assess the potential influence of over-adjustment. Fourth, multi-collinearity was assessed using variance inflation factors. Fifth, because WMH burden, hippocampal volume, and cognitive performance may represent correlated domains of brain aging, an exploratory integrated model was fitted with CES-D score as the dependent variable and the three brain aging markers included simultaneously as predictors to examine the association pattern when these markers were considered together. The model was additionally adjusted for intracranial volume.

Restricted cubic spline models were used to explore potential nonlinearity in the association between CES-D score and WMH burden. Knots were placed at the 5th, 35th, 65th, and 95th percentiles of the CES-D distribution. Nonlinearity was evaluated by testing the spline terms beyond the linear component.

All statistical analyses were performed using Stata version 17 (StataCorp LLC, College Station, TX, United States). A two-sided *p*-value <0.05 was considered statistically significant.

## Results

### Participant characteristics

A total of 2,746 participants with complete data on depressive symptoms, neuroimaging measures, cognitive assessment, and covariates were included in the analytic sample. The median WMH volume was 2657.4 mm^3^ (interquartile range 1491.6–5662.8 mm^3^), and the mean ln-transformed WMH value was 8.00 (SD 0.95). The mean age was 67.5 years (SD 6.5), and 36.3% of participants were men. In the analytic sample, 544 participants (19.8%) had CES-D scores ≥16.

Baseline characteristics according to quartiles of the CES-D total score are presented in Table 1. Participants in higher CES-D quartiles were older and had fewer years of education compared with those in the lowest CES-D quartile (both *p* < 0.001). The proportion of men decreased across CES-D quartiles, and the proportion of participants who were married was lower in higher quartiles (both *p* < 0.001). No statistically significant differences were observed across CES-D quartiles for BMI, SBP, smoking status, or alcohol consumption.

**Table 1:** Baseline characteristics according to quartiles of CES-D total score (*n* = 2,746).

Characteristic	Q1 (≤2) *n* = 783	Q2 (3–6) *n* = 623	Q3 (7–13) *n* = 673	Q4 (≥14) *n* = 667	*p*-value
Age, years	66.86 (6.64)	66.98 (6.26)	67.27 (6.32)	68.81 (6.36)	<0.001
Education, years	10.37 (4.39)	9.67 (4.36)	9.74 (4.02)	8.27 (4.04)	<0.001
BMI, kg/m^2^	24.93 (3.00)	24.77 (3.12)	24.62 (2.86)	24.88 (3.28)	0.239
SBP (min), mmHg	127.05 (15.24)	126.14 (16.26)	126.54 (15.65)	127.87 (15.35)	0.216
Male, %	42.53	35.31	34.77	31.33	<0.001
Married, %	88.76	87.80	86.18	80.51	<0.001
Smoking status, %					0.904
Never smoker	91.95	91.81	92.57	91.30	
Former smoker	2.55	1.93	2.38	2.85	
Current smoker	5.49	6.26	5.05	5.85	
Alcohol consumption, %					0.160
None	63.47	62.76	68.20	66.12	
≤Once/week	19.92	20.71	19.47	20.69	
>Once/week	16.60	16.53	12.33	13.19	
Brain aging markers					
WMH volume, mm^3^, median (IQR)	2282.4 (1386.0–4642.8)	2389.2 (1417.2–5128.8)	2833.2 (1544.4–6090.0)	3181.2 (1720.8–6927.6)	<0.001
Hippocampal volume, mm^3^	6120.85 (710.34)	6106.15 (715.25)	6100.77 (701.82)	5905.04 (727.54)	<0.001
Cognitive performance, SNSB-C T-score	51.19 (11.19)	51.22 (10.73)	49.91 (10.37)	47.62 (10.26)	<0.001

WMH, white matter hyperintensity; CES-D, Center for Epidemiologic Studies Depression Scale; BMI, body mass index; SBP, systolic blood pressure; SNSB-C, Seoul Neuropsychological Screening Battery-Core; IQR, interquartile range. Values are mean (SD) or percentage unless otherwise indicated. WMH volume is presented as median (IQR). *p*-values for continuous variables presented as mean (SD) were obtained using one-way analysis of variance; *p*-value for WMH volume was obtained using the Kruskal–Wallis test; *p*-values for categorical variables were obtained using Pearson’s Chi-square tests. CES-D quartiles were defined using sample-based cut-points of 2, 6, and 13.

Brain aging markers also differed across CES-D quartiles. Median WMH volume increased with higher CES-D quartiles, whereas hippocampal volume and cognitive performance decreased across quartiles (all *p* < 0.001).

### Association between CES-D and WMH burden

Higher CES-D scores were associated with greater WMH burden (Table 2). In the fully adjusted model, each 1-point increase in CES-D score was associated with higher ln-transformed WMH volume (*β* = 0.0073; 95% CI 0.0036–0.0109).

**Table 2:** Association of CES-D with multiple markers of brain aging (*n* = 2,746).

Outcome	Model 1	Model 2	Model 3
Log-transformed WMH volume			
CES-D total score (per 1-point increase)	0.0080 (0.0044–0.0116)***	0.0073 (0.0036–0.0110)***	0.0073 (0.0036–0.0109)***
CES-D quartiles (ref: Q1)			
Q2 vs. Q1	0.0728 (−0.0147–0.1603)	0.0681 (−0.0192–0.1553)	0.0730 (−0.0144–0.1604)
Q3 vs. Q1	0.1647 (0.0786–0.2507)***	0.1608 (0.0747–0.2469)***	0.1638 (0.0779–0.2496)***
Q4 vs. Q1	0.2290 (0.1370–0.3209)***	0.2128 (0.1192–0.3065)***	0.2160 (0.1226–0.3093)***
Hippocampal volume (mm^3^)			
CES-D total score (per 1-point increase)	−4.31 (−6.81–−1.81)***	−3.00 (−5.54–−0.47)*	−3.63 (−6.05–−1.21)**
CES-D quartiles (ref: Q1)			
Q2 vs. Q1	5.23 (−58.78–69.24)	15.63 (−47.57–78.82)	28.25 (−30.02–86.53)
Q3 vs. Q1	17.03 (−46.42–80.48)	24.55 (−38.56–87.67)	15.95 (−43.58–75.49)
Q4 vs. Q1	−88.27 (−153.80–−22.74)**	−57.75 (−123.57–8.06)	−64.26 (−127.17–−1.35)*
Cognitive performance (SNSB-C T-score)			
CES-D total score (per 1-point increase)	−0.17 (−0.21–−0.13)***	−0.16 (−0.20–−0.11)***	−0.16 (−0.20–−0.11)***
CES-D quartiles (ref: Q1)			
Q2 vs. Q1	0.02 (−1.13–1.17)	0.14 (−1.01–1.29)	0.07 (−1.08–1.22)
Q3 vs. Q1	−1.27 (−2.38–−0.16)*	−1.19 (−2.30–−0.09)*	−1.24 (−2.34–−0.13)*
Q4 vs. Q1	−3.48 (−4.60–−2.36)***	−3.17 (−4.29–−2.04)***	−3.20 (−4.34–−2.07)***

CES-D, Center for Epidemiologic Studies Depression Scale; WMH, white matter hyperintensity; SNSB-C, Seoul Neuropsychological Screening Battery-Core; Ref, reference. Values are β coefficients (95% confidence intervals) from linear regression models with robust standard errors. Model 1 was adjusted for age and sex; Model 2 was additionally adjusted for education (years) and marital status; Model 3 was further adjusted for body mass index, systolic blood pressure (minimum of two measurements), smoking status, and alcohol consumption. Hippocampal volume models were additionally adjusted for intracranial volume. For log-transformed WMH volume, overall quartile test (Model 3, Wald test): *p* < 0.001; test for linear trend (Model 3): *p* < 0.001. For hippocampal volume, overall quartile test (Model 3, Wald test): *p* = 0.027; test for linear trend (Model 3): *p* = 0.062. For cognitive performance, overall quartile test (Model 3, Wald test): *p* < 0.001; test for linear trend (Model 3): *p* < 0.001; *p < 0.05; **p < 0.01; ***p < 0.001.

When CES-D scores were analyzed categorically, participants in the third and highest CES-D quartiles had significantly greater WMH burden than those in the lowest quartile (Q3: *β* = 0.1638; 95% CI 0.0779–0.2496; *β* = 0.2160; 95% CI 0.1226–0.3093), whereas the association for the second quartile was not statistically significant.

**Figure 2:**
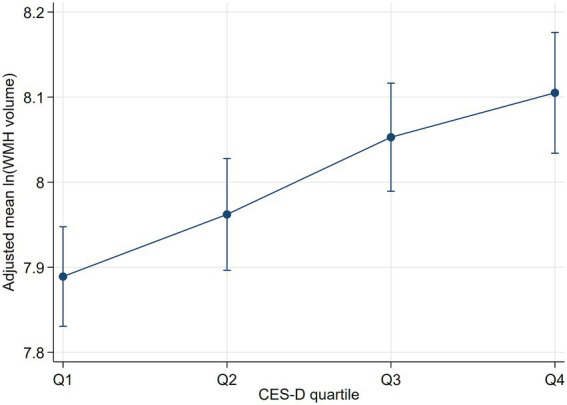
Adjusted mean log-transformed WMH volume across quartiles of the CES-D total score. CES-D, Center for Epidemiologic Studies Depression Scale; WMH, white matter hyperintensity. Estimates were derived from multivariable linear regression adjusted for age, sex, education (years), marital status, body mass index, systolic blood pressure (minimum of two measurements), smoking status, and alcohol consumption in the analytic sample (*n* = 2,746). Error bars indicate 95% confidence intervals.

Overall differences across CES-D quartiles were significant (*p* < 0.001), and a significant linear trend was observed (*p*-for trend <0.001). Adjusted mean ln-transformed WMH values across CES-D quartiles are shown in Figure 2.

Restricted cubic spline analysis suggested nonlinearity in the association between CES-D score and WMH burden (*p*-for nonlinearity = 0.023; Supplementary Figure S1). The positive association appeared more pronounced at lower-to-moderate CES-D scores and flattened at higher CES-D scores.

### Associations with hippocampal volume and cognitive performance

Associations of CES-D with hippocampal volume and cognitive performance are also presented in Table 2. Higher CES-D scores were associated with smaller hippocampal volume after multivariable adjustment. In the fully adjusted model, each 1-point increase in CES-D score was associated with smaller total hippocampal volume (*β* = −3.63 mm^3^; 95% CI −6.05–−1.21). When analyzed categorically, participants in the highest CES-D quartile had smaller hippocampal volume than those in the lowest quartile (*β* = −64.26 mm^3^; 95% CI −127.17–−1.35), whereas differences for the intermediate quartiles were not statistically significant. Overall differences across CES-D quartiles were significant (*p* = 0.027), but the linear trend across quartiles did not reach statistical significance (*p*-for trend = 0.062).

Higher depressive symptom burden was also associated with poorer cognitive performance. In the fully adjusted model, each 1-point increase in CES-D score was associated with a lower SNSB-C total T-score (*β* = −0.16; 95% CI −0.20–−0.11). Consistent with this pattern, participants in the third and highest CES-D quartiles had lower cognitive scores than those in the lowest quartile (Q3: *β* = −1.24; 95% CI −2.34–−0.13; Q4: *β* = −3.20; 95% CI −4.34–−2.07), whereas the association for the second quartile was not statistically significant. Overall differences across CES-D quartiles and the linear trend across quartiles were both significant (both *p* < 0.001).

**Figure 3:**
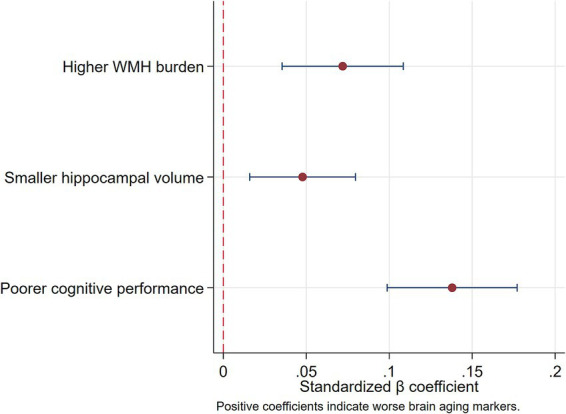
Standardized associations between depressive symptoms and multiple markers of brain aging. WMH, white matter hyperintensity. Points indicate standardized *β* coefficients and horizontal lines indicate 95% confidence intervals from fully adjusted linear regression models estimated in the analytic sample (*n* = 2,746). For visualization purposes, coefficients for hippocampal volume and cognitive performance were directionally reversed so that higher values consistently reflect worse brain aging markers. Models were adjusted for age, sex, education (years), marital status, body mass index, systolic blood pressure (minimum of two measurements), smoking status, and alcohol consumption. The hippocampal volume model was additionally adjusted for intracranial volume.

Standardized associations between CES-D score and the three brain aging markers are summarized in Figure 3. In this analysis, higher CES-D scores were associated with higher WMH burden, smaller hippocampal volume, and poorer cognitive performance, with the largest standardized association observed for poorer cognitive performance.

### Sensitivity analyses

Additional adjustment for vascular comorbidities, including hypertension, diabetes mellitus, and dyslipidemia, did not materially alter the associations of CES-D with WMH burden or cognitive performance (Supplementary Table S1). The association with hippocampal volume remained evident for continuous CES-D score, although the categorical association for the highest CES-D quartile was attenuated after additional vascular comorbidity adjustment.

When depressive symptoms were modeled as a dichotomous exposure using a CES-D cutoff of ≥16, clinically relevant depressive symptom burden was associated with greater WMH burden, smaller hippocampal volume, and poorer cognitive performance (Supplementary Table S2). Cognitive performance models excluding age and education from covariate adjustment produced results consistent with the main analysis (Supplementary Table S3). Multi-collinearity diagnostics showed no evidence of problematic collinearity, with all variance inflation factors below 2.5 (Supplementary Table S4).

In the exploratory integrated model, log-transformed WMH volume, hippocampal volume, and cognitive performance were considered simultaneously in relation to CES-D score. In this model, higher WMH burden and poorer cognitive performance remained associated with higher CES-D score, whereas hippocampal volume did not show a statistically significant association with CES-D score (Supplementary Table S5).

## Discussion

In this population-based cohort of community-dwelling older adults, greater depressive symptom burden was associated with multiple markers of brain aging, including higher WMH volume, smaller hippocampal volume, and poorer cognitive performance. These associations were observed after adjustment for demographic, cardio-metabolic, and lifestyle factors. The findings were most consistent for WMH burden and cognitive performance, whereas the association with hippocampal volume was more modest. Taken together, these results suggest that depressive symptoms in later life are related to multiple domains relevant to brain aging, although the strength and consistency of these associations may differ across vascular, structural, and cognitive domains.

The association between depressive symptoms and greater WMH burden observed in this study is consistent with prior evidence linking late-life depressive symptoms to cerebral small vessel disease (Herrmann et al., 2008; Taylor et al., 2013; Alexopoulos et al., 1997). According to the vascular depression hypothesis, cerebrovascular pathology may disrupt fronto–subcortical circuits involved in mood regulation, thereby increasing vulnerability to depressive symptoms in older adults (Taylor et al., 2013; Alexopoulos et al., 1997). Population-based neuroimaging studies have similarly reported associations between WMH burden and depressive symptoms in older adults, supporting the relevance of vascular mechanisms in late-life depressive symptoms (Taylor et al., 2013; de Groot et al., 2000; Geraets et al., 2021). In the present study, the association between depressive symptoms and WMH burden remained evident after adjustment for demographic, cardio-metabolic, and lifestyle factors, as well as after additional adjustment for vascular comorbidities, suggesting that the observed relationship was not fully explained by measured vascular comorbidities.

Restricted cubic spline analysis suggested modest nonlinearity in the association between CES-D score and WMH burden. The positive association appeared more pronounced at lower-to-moderate CES-D scores and flattened at higher CES-D scores. Prior late-life depression neuroimaging studies have often relied on linear or categorical models, and several population-based studies have suggested threshold-like or heterogeneous patterns according to lesion burden, age group, or symptom severity (de Groot et al., 2000; Geraets et al., 2021). Thus, the spline findings in the present study suggest that the association between depressive symptoms and WMH burden may not be uniform across the full range of depressive symptom severity.

Depressive symptoms were also associated with smaller hippocampal volume, although the magnitude and consistency of this association were more modest than those observed for WMH burden and cognitive performance. The hippocampus is particularly vulnerable to stress-related neurobiological processes, including prolonged glucocorticoid exposure and neuro-inflammation (McEwen, 2007; Otte et al., 2016; Sapolsky, 2000; Miller and Raison, 2016). Prior studies have reported that associations between depressive symptoms and hippocampal volume in older adults are often heterogeneous, particularly in community-based cohorts and in individuals with relatively mild or subclinical depressive symptoms (Sexton et al., 2013; Monereo-Sánchez et al., 2024). In the present study, significant group differences in hippocampal volume were observed mainly between participants in the highest and lowest CES-D quartiles, whereas differences for the intermediate quartiles were not statistically significant. The categorical association for the highest CES-D quartile was attenuated after additional adjustment for vascular comorbidities. These findings may suggest that hippocampal volume is a heterogeneous correlate of depressive symptoms in older adults and may be less consistently detectable in community-based populations with generally mild depressive symptom burden.

Greater depressive symptom burden was also associated with poorer global cognitive performance in this study. This finding is consistent with previous epidemiological studies reporting associations between late-life depressive symptoms, lower cognitive function, and increased risk of cognitive decline and dementia (Livingston et al., 2020; Diniz et al., 2013; Ownby et al., 2006; Wang et al., 2024). Because global cognitive performance reflects the integrated functioning of multiple neural systems, depressive symptoms in older adults may be associated with broader differences in brain function relevant to aging.

Taken together, the pattern of findings suggests that depressive symptoms in later life are related to multiple, partially overlapping domains of brain aging rather than to a single isolated marker. The exploratory integrated model provided complementary evidence regarding the relative consistency of the observed associations when multiple brain aging markers were considered simultaneously. In this model, WMH burden and cognitive performance remained associated with depressive symptom burden, whereas hippocampal volume did not show a statistically significant association. This exploratory pattern suggests that vascular brain changes and cognitive performance may show more consistent associations with depressive symptoms in community-dwelling older adults, while hippocampal volume may capture a more heterogeneous or less proximal component of brain aging. However, because the integrated model was exploratory and the study design was cross-sectional, these findings should not be interpreted as evidence of directionality among depressive symptoms, brain structural markers, and cognitive performance.

Several interacting biological mechanisms may underlie the observed associations between depressive symptoms and multiple domains of brain aging. Cerebrovascular pathology related to small vessel disease may contribute to disruption of fronto–subcortical circuits involved in mood regulation (Taylor et al., 2013; Alexopoulos et al., 1997), while stress-related neurobiological processes, including prolonged glucocorticoid exposure and neuro-inflammation, may be associated with hippocampal structural changes (McEwen, 2007; Otte et al., 2016; Sapolsky, 2000; Miller and Raison, 2016). In addition, shared vascular and metabolic risk factors, such as hypertension and other cardio-metabolic conditions, may influence both depressive symptoms and brain integrity. These mechanisms are unlikely to operate independently and may instead interact to influence multiple aspects of brain aging in later life.

This study has several strengths. First, we evaluated multiple markers of brain aging, including WMH burden, hippocampal volume, and cognitive performance, within the same population-based cohort of community-dwelling older adults. This approach allowed comparison of vascular, structural, and cognitive domains in the same analytic sample. Second, all neuroimaging measures were processed using a centralized MRI quality-control and image-processing pipeline with harmonized acquisition protocols across study sites. Third, the consistency of findings across multiple analytic approaches, including continuous, quartile-based, and clinically relevant CES-D cutoff analyses, supports the robustness of the observed associations. Finally, the relatively large population-based cohort of community-dwelling older adults enhances the relevance of the findings to real-world aging populations.

Several limitations should be considered. First, the cross-sectional design precludes conclusions regarding temporal or causal relationships between depressive symptoms and the observed neuroimaging markers and cognitive performance. Depressive symptoms, cognitive performance, and structural brain changes in later life may be bi-directionally related or may reflect shared underlying processes, which could not be disentangled in the present cross-sectional analysis. Second, depressive symptoms were assessed using a self-report scale rather than a clinical diagnostic interview, which may introduce measurement error or misclassification. Third, although we adjusted for demographic, cardio-metabolic, and lifestyle factors, residual confounding from unmeasured variables cannot be excluded. Fourth, follow-up data from the ongoing CAVAS-CA cohort were not yet available at the time of analysis, limiting the ability to evaluate longitudinal trajectories or temporal relationships between depressive symptoms and brain aging markers. Finally, because the study population consisted of community-dwelling older adults in South Korea, the findings may not be fully generalizable to other populations.

These findings may have implications for understanding the clinical relevance of depressive symptoms in aging populations. Even in community-dwelling older adults with generally mild depressive symptom burden, depressive symptoms were associated with multiple markers relevant to brain aging, particularly WMH burden and cognitive performance. This pattern suggests that depressive symptoms in later life may reflect broader vulnerability related to vascular and cognitive aspects of brain aging. Accordingly, assessment of depressive symptoms in older adults may provide clinically relevant information regarding brain aging-related vulnerability.

In conclusion, greater depressive symptom burden was associated with higher WMH burden, smaller hippocampal volume, and poorer cognitive performance among community-dwelling older adults. These associations were most consistent for WMH burden and cognitive performance, whereas the association with hippocampal volume was more modest. The findings suggest that depressive symptoms in later life are related to multiple domains of brain aging and support the relevance of evaluating depressive symptoms in the broader context of vascular, structural, and cognitive domains of brain aging in older adults. Longitudinal studies with repeated assessments of depressive symptoms, neuroimaging markers, and cognitive performance will be important for clarifying temporal relationships and trajectories of brain aging in later life.

## Data Availability

The datasets presented in this article are not readily available because data access is restricted due to cohort data access policies, and datasets are available from the corresponding authors upon reasonable request and subject to approval by the KoGES CAVAS-CA data access committee. Requests to access the datasets should be directed to JH, imitate2017@gmail.com.
