# Supplementary material for: Depressive symptoms and multiple markers of brain aging in community-dwelling older adults
Source: Front Aging Neurosci. 2026 Jun 23;18:1868746. doi: 10.3389/fnagi.2026.1868746 (PMC13337943; doi:10.3389/fnagi.2026.1868746)
Supplement: Supplementary file 1 [file Data_Sheet_1.PDF]

Supplementary material captions

Supplementary Table S1. Association of CES-D with WMH burden, hippocampal volume, and cognitive performance after additional adjustment for vascular comorbidities

Supplementary Table S2. Association of CES-D clinical cutoff with WMH burden, hippocampal volume, and cognitive performance

Supplementary Table S3. Association of CES-D with cognitive performance after excluding age and education from covariate adjustment

Supplementary Table S4. Variance inflation factors for fully adjusted regression models

Supplementary Table S5. Exploratory integrated model including multiple brain aging markers in relation to CES-D score

Supplementary Figure S1. Restricted cubic spline analysis of CES-D score and log-transformed WMH volume

Supplementary material

Supplementary Table S1. Association of CES-D with WMH burden, hippocampal volume, and cognitive performance after additional adjustment for vascular comorbidities

Outcome	β (95% CI)
**Log-transformed WMH volume**	
CES-D total score (per 1-point increase)	0.0071 (0.0034–0.0108)***
CES-D quartiles (Ref: Q1)	
Q2 vs Q1	0.0718 (−0.0157–0.1592)
Q3 vs Q1	0.1612 (0.0756–0.2468)***
Q4 vs Q1	0.2128 (0.1192–0.3064)***
**Hippocampal volume (mm³)**	
CES-D total score (per 1-point increase)	−3.54 (−5.97–−1.11)**
CES-D quartiles (Ref: Q1)	
Q2 vs Q1	28.72 (−29.56–86.99)
Q3 vs Q1	16.86 (−42.69–76.42)
Q4 vs Q1	−62.66 (−125.74–0.42)
**Cognitive performance (SNSB-C T-score)**	
CES-D total score (per 1-point increase)	−0.15 (−0.20–−0.11)***
CES-D quartiles (Ref: Q1)	
Q2 vs Q1	0.05 (−1.09–1.20)
Q3 vs Q1	−1.22 (−2.32–−0.11)*
Q4 vs Q1	−3.13 (−4.27–−2.00)***

Abbreviations: CES-D, Center for Epidemiologic Studies Depression Scale; WMH, white matter hyperintensity; SNSB-C, Seoul Neuropsychological Screening Battery-Core; Ref, reference. Values are β coefficients (95% confidence intervals) from linear regression models with robust standard errors. Models were adjusted for age, sex, education (years), marital status, body mass index, systolic blood pressure (minimum of two measurements), smoking status, alcohol consumption, hypertension, diabetes mellitus, and dyslipidemia. Hippocampal volume models were additionally adjusted for intracranial volume. For log-transformed WMH volume, overall quartile test: $p < 0.001$; test for linear trend: $p < 0.001$. For hippocampal volume, overall quartile test: $p = 0.031$; test for linear trend: $p = 0.070$. For cognitive performance, overall quartile test: $p < 0.001$; test for linear trend: $p < 0.001$. *$p < 0.05$; **$p < 0.01$; ***$p < 0.001$.

Supplementary Table S2. Association of CES-D clinical cutoff with WMH burden, hippocampal volume, and cognitive performance

Outcome	β (95% CI)
Log-transformed WMH volume	0.1038 (0.0149–0.1926)*
Hippocampal volume (mm^3)	−71.50 (−129.54–−13.46)*
Cognitive performance (SNSB-C T-score)	−2.82 (−3.84–−1.79)***

Abbreviations: CES-D, Center for Epidemiologic Studies Depression Scale; WMH, white matter hyperintensity; SNSB-C, Seoul Neuropsychological Screening Battery-Core. Values are β coefficients (95% confidence intervals) for CES-D ≥16 versus CES-D <16 from fully adjusted linear regression models with robust standard errors. Models were adjusted for age, sex, education (years), marital status, body mass index, systolic blood pressure (minimum of two measurements), smoking status, and alcohol consumption. The hippocampal volume model was additionally adjusted for intracranial volume. *$p < 0.05$; **$p < 0.01$; ***$p < 0.001$.

Supplementary Table S3. Association of CES-D with cognitive performance after excluding age and education from covariate adjustment

Outcome	β (95% CI)
**Cognitive performance (SNSB-C T-score)**	
CES-D total score (per 1-point increase)	−0.17 (−0.21–−0.13)***
CES-D quartiles (Ref: Q1)	
Q2 vs Q1	−0.05 (−1.21–1.10)
Q3 vs Q1	−1.33 (−2.44–−0.23)*
Q4 vs Q1	−3.56 (−4.68–−2.44)***
CES-D ≥16 vs <16	−3.13 (−4.14–−2.12)***

Abbreviations: CES-D, Center for Epidemiologic Studies Depression Scale; SNSB-C, Seoul Neuropsychological Screening Battery-Core; Ref, reference. Values are β coefficients (95% confidence intervals) from linear regression models with robust standard errors. Models were adjusted for sex, marital status, body mass index, systolic blood pressure (minimum of two measurements), smoking status, and alcohol consumption, excluding age and education because the SNSB-C T-score is standardized by age and education. Overall quartile test: $p < 0.001$; test for linear trend: $p < 0.001$. *$p < 0.05$; **$p < 0.01$; ***$p < 0.001$.

Supplementary Table S4. Variance inflation factors for fully adjusted regression models

Variable	WMH model VIF	Hippocampal volume model VIF	Cognitive performance model VIF
CES-D total score	1.05	1.05	1.05
Age	1.29	1.30	1.29
Sex	1.62	2.20	1.62
Education	1.31	1.32	1.31
Marital status	1.11	1.11	1.11
BMI	1.06	1.06	1.06
SBP	1.09	1.09	1.09
Smoking status	1.05–1.13	1.06–1.13	1.05–1.13
Alcohol consumption	1.14–1.33	1.14–1.33	1.14–1.33
Intracranial volume	—	1.65	—
**Mean VIF**	**1.20**	**1.29**	**1.20**

Abbreviations: WMH, white matter hyperintensity; BMI, body mass index; SBP, systolic blood pressure; VIF, variance inflation factor. Variance inflation factors were calculated using fully adjusted regression models estimated in the unified analytic sample (n = 2,746). All VIF values were below 2.5, indicating no evidence of problematic multicollinearity.

Supplementary Table S5. Exploratory integrated model including multiple brain aging markers in relation to CES-D score

Predictor	β (95% CI)	Standardized β (95% CI)
Log-transformed WMH volume	0.5759 (0.1306–1.0211)*	0.0581 (0.0132–0.1031)*
Hippocampal volume (mm³)	−0.0005 (−0.0011–0.0002)	−0.0359 (−0.0851–0.0133)
Cognitive performance (SNSB-C T-score)	−0.1074 (−0.1419–−0.0730)***	−0.1222 (−0.1614–−0.0831)***

Abbreviations: CES-D, Center for Epidemiologic Studies Depression Scale; WMH, white matter hyperintensity; SNSB-C, Seoul Neuropsychological Screening Battery-Core. Values are β coefficients (95% confidence intervals) from an exploratory linear regression model with robust standard errors in which CES-D total score was modeled as the dependent variable. Because WMH burden, hippocampal volume, and cognitive performance may represent correlated domains of brain aging, the model included these markers simultaneously to examine the association pattern when considered together. The model was adjusted for age, sex, education (years), marital status, body mass index, systolic blood pressure (minimum of two measurements), smoking status, alcohol consumption, and intracranial volume. *$p < 0.05$; **$p < 0.01$; ***$p < 0.001$.

Supplementary Figure S1. Restricted cubic spline analysis of CES-D score and log-transformed WMH volume

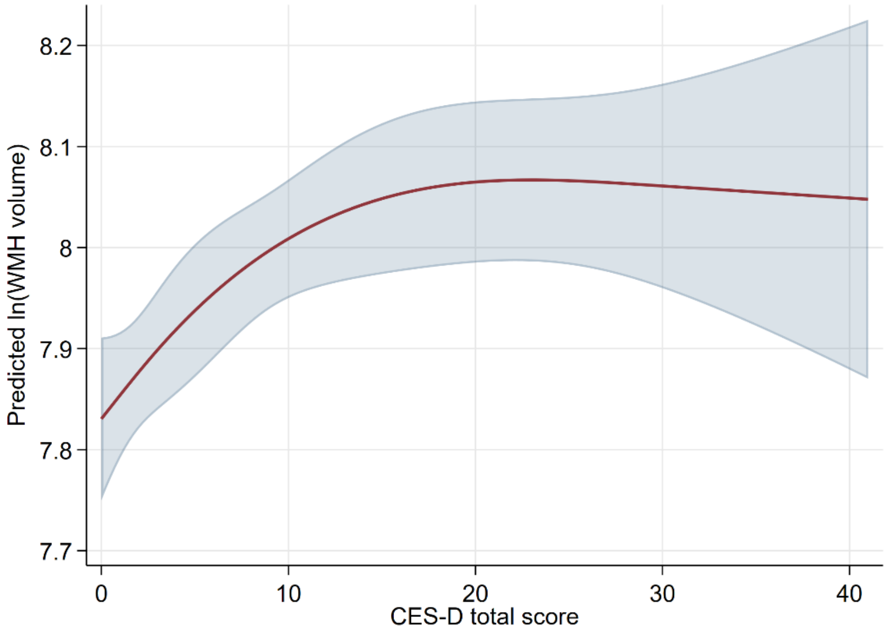

Abbreviations: CES-D, Center for Epidemiologic Studies Depression Scale; WMH, white matter hyperintensity. Restricted cubic spline model showing the association between CES-D score and log-transformed WMH volume. Knots were placed at the 5th, 35th, 65th, and 95th percentiles of the CES-D distribution (0, 3, 10, and 29, respectively). The solid line represents the estimated association from a multivariable linear regression model adjusted for age, sex, education (years), marital status, body mass index, systolic blood pressure (minimum of two measurements), smoking status, and alcohol consumption; shaded areas indicate 95% confidence intervals. Evidence of nonlinearity was observed (p for nonlinearity = 0.023), with the positive association appearing more pronounced at lower-to-moderate CES-D scores and flattening at higher CES-D scores.
